# A Multi-Center Randomized Trial to Assess the Efficacy of Gatifloxacin versus Ciprofloxacin for the Treatment of Shigellosis in Vietnamese Children

**DOI:** 10.1371/journal.pntd.0001264

**Published:** 2011-08-02

**Authors:** Ha Vinh, Vo Thi Cuc Anh, Nguyen Duc Anh, James I. Campbell, Nguyen Van Minh Hoang, Tran Vu Thieu Nga, Nguyen Thi Khanh Nhu, Pham Van Minh, Cao Thu Thuy, Pham Thanh Duy, Le Thi Phuong, Ha Thi Loan, Mai Thu Chinh, Nguyen Thi Thu Thao, Nguyen Thi Hong Tham, Bui Li Mong, Phan Van Be Bay, Jeremy N. Day, Christiane Dolecek, Nguyen Phu Huong Lan, To Song Diep, Jeremy J. Farrar, Nguyen Van Vinh Chau, Marcel Wolbers, Stephen Baker

**Affiliations:** 1 The Hospital for Tropical Diseases, Ho Chi Minh City, Vietnam; 2 The Hospital for Tropical Diseases, Wellcome Trust Major Overseas Programme, Oxford University Clinical Research Unit, Ho Chi Minh City, Vietnam; 3 Huu Nghi Hospital, Cao Lanh, Dong Thap Province, Vietnam; 4 Centre for Tropical Medicine, Nuffield Department of Clinical Medicine, Oxford University, Oxford, United Kingdom; 5 School of Medicine and Pharmacy, Ho Chi Minh City, Vietnam; Fondation Raoul Follereau, France

## Abstract

**Background:**

The bacterial genus *Shigella* is the leading cause of dysentery. There have been significant increases in the proportion of *Shigella* isolated that demonstrate resistance to nalidixic acid. While nalidixic acid is no longer considered as a therapeutic agent for shigellosis, the fluoroquinolone ciprofloxacin is the current recommendation of the World Health Organization. Resistance to nalidixic acid is a marker of reduced susceptibility to older generation fluoroquinolones, such as ciprofloxacin. We aimed to assess the efficacy of gatifloxacin versus ciprofloxacin in the treatment of uncomplicated shigellosis in children.

**Methodology/Principal Findings:**

We conducted a randomized, open-label, controlled trial with two parallel arms at two hospitals in southern Vietnam. The study was designed as a superiority trial and children with dysentery meeting the inclusion criteria were invited to participate. Participants received either gatifloxacin (10 mg/kg/day) in a single daily dose for 3 days or ciprofloxacin (30 mg/kg/day) in two divided doses for 3 days. The primary outcome measure was treatment failure; secondary outcome measures were time to the cessation of individual symptoms. Four hundred and ninety four patients were randomized to receive either gatifloxacin (n  =  249) or ciprofloxacin (n  =  245), of which 107 had a positive *Shigella* stool culture. We could not demonstrate superiority of gatifloxacin and observed similar clinical failure rate in both groups (gatifloxacin; 12.0% and ciprofloxacin; 11.0%, *p*  =  0.72). The median (inter-quartile range) time from illness onset to cessation of all symptoms was 95 (66–126) hours for gatifloxacin recipients and 93 (68–120) hours for the ciprofloxacin recipients (Hazard Ratio [95%CI]  =  0.98 [0.82–1.17], *p*  =  0.83).

**Conclusions:**

We conclude that in Vietnam, where nalidixic acid resistant *Shigellae* are highly prevalent, ciprofloxacin and gatifloxacin are similarly effective for the treatment of acute shigellosis.

**Trial Registration:**

Controlled trials number ISRCTN55945881

## Author Summary

The bacterial genus *Shigella* is the most common cause of dysentery (diarrhea containing blood and/or mucus) and the disease is common in developing countries with limitations in sanitation. Children are most at risk of infection and frequently require hospitalization and antimicrobial therapy. The WHO currently recommends the fluoroquinolone, ciprofloxacin, for the treatment of childhood *Shigella* infections. In recent years there has been a sharp increase in the number of organisms that exhibit resistance to nalidixic acid (an antimicrobial related to ciprofloxacin), corresponding with reduced susceptibility to ciprofloxacin. We hypothesized that infections with *Shigella* strains that demonstrate resistance to nalidixic acid may prevent effective treatment with ciprofloxacin. We performed a randomized controlled trial to compare 3 day ciprofloxacin therapy with 3 days of gatifloxacin, a newer generation fluoroquinolone with greater activity than ciprofloxacin. We measured treatment failure and time to the cessation of individual disease symptoms in 249 children with dysentery treated with gatifloxacin and 245 treated with ciprofloxacin. We could identify no significant differences in treatment failure between the two groups or in time to the cessation of individual symptoms. We conclude that, in Vietnam, ciprofloxacin and gatifloxacin are similarly effective for the treatment of acute dysentery.

## Introduction

Dysentery is an infection of the gastrointestinal tract characterized by diarrhea containing blood and/or mucous, abdominal cramping and tenesmus. The major cause of dysentery is the bacterial genus *Shigella*. Humans are the only known reservoir of *Shigella*, which are transmitted from person to person by the fecal-oral route, the infectious dose is low [1], and children under five years of age are at the greatest risk of infection [2]. The precise burden of Shigellosis is difficult to estimate, particularly in developing countries, as many cases go unreported and accurate diagnosis relies on bacterial culture, which is not always feasible or reliable. World Health Organization (WHO) estimates in 1999 put the number of cases at 164.7 million per year worldwide, with 99% of these infections occurring in developing countries [3]. The WHO also estimated that Shigellosis was responsible for over a million deaths per year [4]. *Shigella* are endemic in Vietnam [5], and in Nha Trang, in central Vietnam, an incidence of 490/100,000 in children under five has been estimated [6].

Antimicrobials are routinely administered to Shigellosis patients and correspond with clinical improvement [7], [8], however, many infecting organisms from various regions are now resistant to multiple antimicrobials [9]–[11]. In southern Vietnam we have recently documented a transition from *S. flexneri* to *S. sonnei* which has been concurrent with a dramatic shift in antimicrobial resistance patterns [12]. The majority (> 90%) of *Shigella* strains we now isolate demonstrate resistance to nalidixic acid [12]. Whilst nalidixic acid is no longer considered as an effective therapeutic agent for shigellosis, the fluoroquinolone, ciprofloxacin, is the current recommendation of the WHO [4], [13]. Ciprofloxacin is considered to be efficacious, cost effective and generally safe for use in a pediatric population. However, resistance to nalidixic acid generally correlates with a decreased susceptibility to ciprofloxacin and other older generation fluoroquinolones [14]. Mutations within the DNA gyrase gene (*gyrA*), the topoisomerase gene (*parC*) and multiple plasmid mediated quinolone resistance (PMQR) determinants can elevate the minimum inhibitory concentration (MIC) to ciprofloxacin, and may hinder effective treatment [15]. In fact, some *Shigella* strains that have been isolated from patients with dysentery have already been reported as being resistant to ciprofloxacin, with an MIC > 0.25 µg/mL [16]. Alternative regimens are obviously needed for those patients infection with organisms that may exhibit resistance to ciprofloxacin.

Gatifloxacin is a fourth generation fluoroquinolone. It has a broad spectrum of activity, with potent activity against many gram-positive and gram-negative organisms [17]. Gatifloxacin has been shown to be able be distributed extensively throughout human tissues and can actively penetrate phagocytic cells *in vitro* [18]. Significant intracellular accumulation means that gatifloxacin demonstrates bactericidal activity against susceptible intracellular pathogens, such as *Shigella* [19]. Gatifloxacin has been demonstrated to be highly efficacious in the treatment of enteric fever caused by nalidixic acid resistant organisms in trials performed in Nepal and Vietnam [20], [21]. A differential binding motif of gatifloxacin to the DNA gyrase (*gyrA*) with respect to ciprofloxacin, means that this agent is less prone to the inhibition induced by the common resistance associated mutations [15].

To date there very few published trials studying the efficacy of fluoroquinolones in the treatment of shigellosis in children [22]–[24] and we hypothesized that a therapy with gatifloxacin would be effective in treating shigellosis, particularly in an area with a high level of nalidixic acid resistance. We conducted a randomized controlled trial (RCT) evaluating the efficacy of gatifloxacin versus ciprofloxacin in pediatric patients with uncomplicated dysentery in two locations in southern Vietnam where nalidixic acid resistant *Shigella* predominate.

## Materials and Methods

### Trial registration

Controlled trials number ISRCTN55945881.

### Ethical approval

This study was conducted according to the principles expressed in the Declaration of Helsinki. This work was approved by the institutional ethical review boards of the Hospital for Tropical Diseases, Ho Chi Minh City, Huu Nghi Hospital, and The Oxford Tropical Research Ethics Committee (OXTREC) (number 010–06 (2006)), United Kingdom. The parent or guardian of all enrollees was required to provide written informed consent prior to entrance into the study.

### Trial design

An open label randomized comparison of gatifloxacin (10 mg/kg/day once-a-day orally) for 3 days versus ciprofloxacin (30 mg/kg/day in twice-a-day orally) for 3 days for the treatment of uncomplicated bacillary dysentery in children. This trial was design to demonstrate the superiority of gatifloxacin over ciprofloxacin.

### Study participants

The study physicians enrolled patients who were admitted to Pediatric Ward B, The Hospital for Tropical Diseases, Ho Chi Minh City, Vietnam or the Department of Infectious Diseases, Huu Nghi Hospital, Cao Lanh, Dong Thap, Vietnam from June 22^nd^, 2006 to March 13^th^, 2009. The study included children under the age of 15 years with a history of passing bloody or mucoid stools, with or without abdominal pain, tenesmus or fever (defined as a temperature >37.8°C) for less than 72 hours prior to admission. Exclusion criteria were any signs of a severe infection, including shock, jaundice, extensive gastrointestinal bleeding, a previous history of hypersensitivity to either of the trial drugs, known previous treatment with any (fluoro) quinolone during the current bout of disease prior to hospital admission, or a coexisting infection requiring antimicrobial therapy. Additionally, all children that had a trophozoite or *Entamoeba histolytica* present in their stool on microscopic examination were excluded from enrolment.

### Study intervention

Each patient recruited into the study was randomized to receive one of two regimens, treatment with either gatifloxacin (Stada pharmaceuticals, Vietnam) 10 mg/kg/day in a single daily dose for 3 days or treatment with ciprofloxacin (OPV manufacturer, Vietnam) 30 mg/kg/d in two divided doses for 3 days. Other treatments, including fluid (ORS, parenteral) and antipyretics (paracetamol) were given to enrollees in both groups according to the treating clinicians' discretion. Seizures were treated with diazepam (OPV manufacturer, Vietnam) 0.25 mg/kg intravenously. No additional anti-diarrheal drugs, including smectite, loperamide or probiotics were permitted or used.

### Study procedures

On admission (study day 0), a detailed history of the present illness was documented on a standard report form which recorded the duration of illness prior to admission to hospital (days), the presence of fever (defined as a prolonged temperature > 37.8°C), abdominal discomfort, vomiting, bloody or mucoid diarrhea (defined as ≥3 loose stools with obvious blood or mucus), estimated number of episodes of diarrhea before attending hospital, convulsions believed to be related to fever and/or infection and the administration of any known pre-treatment with antimicrobials and or other treatment. Baseline data including age (in months), sex, location of residence and weight (kg) were also collected.

A physical examination was performed on admission and daily thereafter until discharge. Particular note was taken of any bone or joint symptoms. The axillary temperature, pulse rate, respiratory rate, blood pressure, and frequency and character of stools were recorded every six hours. A full blood cell count was performed on all patients and stools were examined by microscopy (HPF (× 400)) to identify parasites, and to count white blood cells and red blood cells; the cell counts were scored on scale from zero to three, scale 0  =  0 cells/HPF, scale 1  =  1 to 10 cells/HPF, scale 2  =  11 to 20 cells/HPF, scale 3  =  21 to 30 cells/HPF and scale 4  =  >40 cells/HPF. A blood sample was taken on Day 0 (on enrolment) and on day 3 (at the same time at the blood sample on Day 0) and glucose test was performed on those in the gatifloxacin group to assess any potential dysglycemia (hypoglycemia or hyperglycemia) in this portion of the study population over the duration of the therapy. Time from initial investigation in hospital to the cessation of bloody/mucoid and watery diarrhea (in hours) was recorded. Duration of hospital stay was recorded in days post admission; patients were only discharged when all clinical symptoms had resolved completely.

Enrolled patients were additionally required to attend a follow up visit, which was scheduled at seven days after discharge. During the follow up visit, the parents or guardians of the enrolled were questioned about their general health after discharge. Data regarding shigellosis-related symptoms were recorded in standard report form and a stool sample was collected for microbiological culture.

### Microbiological methods

Patients stool samples were cultured overnight in selenite F broth (Oxoid, Basingstoke, UK) and onto MacConkey and XLD agar (Oxoid) at 37°C. Colonies suggestive of *Salmonella* or *Shigella* (non-lactose fermenting) were sub-cultured on to nutrient agar and were identified using a ‘short set’ of sugar fermentation reactions (Klinger iron agar, urea agar, citrate agar, SIM motility-indole media (Oxoid)). After incubation for 18–24 h at 37°C, the test media were read for characteristic *Shigella* reactions and API 20E test strips of biochemical reactions (Biomerieux, Paris, France) were used to confirm the identity of *Shigella spp*. Serologic identification was performed by slide agglutination with polyvalent somatic (O) antigen grouping sera, followed by testing with available monovalent antisera for specific serotype identification as per the manufacturers recommendations (Denka Seiken, Japan). Antimicrobial susceptibility testing of all *Shigella* isolates against nalidixic acid (NAL), ciprofloxacin (CIP) and gatifloxacin (GAT) was performed by disk diffusion following standardized Clinical and Laboratory Standards Institute methods [25]. The minimum inhibitory concentrations (MICs) were additionally calculated for all isolates by E-test, according to manufacturer's recommendations (AB Biodisk, Solna, Sweden) and were compared to control strain *E. coli* ATCC 25922 and an in house fully sensitive *E. coli* control. Isolates were stored on Protect beads (Prolabs, Oxford, United Kingdom) at −70°C.

### Primary endpoints

The pre-defined primary endpoint of this study was the composite endpoint of in-hospital treatment failure defined as the occurrence of any clinical or microbiological treatment failure (or both). A clinical treatment failure was defined as fever (≥ 37.8°C), or the persistence of any signs or symptoms of the disease. These symptoms were defined as vomiting, abdominal pain or tenesmus with or without ≥3 loose stools with our without blood, mucus or blood and mucus after 120 hours of start of either treatment. Clinical treatment failures were subsequently treated with ceftriaxone at 50 mg/kg/day intravenously in single doses for 3 days at the discretion of the treating physician. A microbiological treatment failure was defined as a positive stool culture for the original infecting pathogen after the antimicrobial course was complete (day 3 of treatment or later).

### Secondary endpoints

The secondary endpoints were defined to describe the time from study enrolment (i.e. from the first dose of study treatment) until resolution of specific symptoms. As Shigellosis can be self-limiting, the time to cessation of all symptoms was additionally calculated from the time of disease onset, i.e. accounting for the duration of disease prior to hospital admission. The secondary endpoints were fever clearance time (FCT), i.e. time until the temperature was for the first time ≤37.8°C and subsequently remained ≤37.8°C for at least 48 hours; bloody diarrhea clearance time (BDCT), i.e. time until the first non-bloody stool recorded; diarrhea clearance time (DCT), i.e. time until the first non-diarrheal stool was recorded; and total duration of illness, i.e. the time till cessation of all symptoms. The follow-up visit was scheduled to occur 7 days after hospital discharge and information at this time point was not deemed to be as quantifiable as outcomes during hospitalization and, therefore, was not included in the primary outcome but the proportion of patients with failure at the follow-up visit (overall combined failure, diarrhea and other symptoms of shigellosis) was reported as a secondary endpoint.

### Sample size

This trial was designed as a superiority trial of gatifloxacin versus ciprofloxacin with the hypothesis that in patients with *Shigella* positive stool culture, gatifloxacin reduces the proportion of patients failing treatment from 25% to 5%. To detect such a reduction with 80% power at the two-sided 5% significance level, each group required 58 culture confirmed enrollees. We estimated the rate of *Shigella* positive stool culture to be approximately 33% of all children with bloody of mucoid diarrhea and the dropout rate to be approximately 5% and thus aimed to enroll a total of 366 cases with acute dysenteric syndrome. However, when 366 cases had been enrolled, only 80 culture positive patients with *Shigella* were identified (culture rate 22%), and the study was extended to enroll 134 more cases (500 in total). When 500 patients had been enrolled, 107 cases were stool culture positive for *Shigella*. Because of time and budget limitations, recruitment was terminated at 500. Of note, the original sample size calculation was based on a conservative sample size formula; a more precise power estimate based on simulation revealed that the power to detect the target effect with the recruited number of *Shigella* patients was 81%.

### Randomization

Patients were randomly allocated to one of two treatments. An administrator otherwise not involved in the trial performed randomization in blocks of 10. The random allocations were placed in sealed opaque envelopes, which were opened once the patient was enrolled into the trial after meeting the inclusion and exclusion criteria. Consecutive patients who presented to the clinic and were eligible to be enrolled were randomized and enrolled in the same sequence as they presented. Blinding was not carried out in this trial due to different frequencies of administration and other logistical reasons.

### Statistical methods

The proportion of patients reaching the primary endpoint of overall treatment failure and its components, respectively, were compared between the two arms using χ^2^-tests (p-values were calculated by Monte Carlo simulation if the expected count in any cell was <5) and 95% confidence intervals for absolute risk differences were calculated according to the method of Agresti and Caffo [26].

We analyzed the secondary time-to-event endpoints using survival methods and comparisons were based on Cox regression models with treatment as the sole covariate. We also summarized the proportion of patients with failure at the follow-up visit as for the primary endpoint. Only patients who attended the follow-up visit were included, i.e. we did not perform any imputation of missing data.

The main analysis population included all patients who received at least one dose of the intervention analyzed according to the randomized treatment group (intention-to-treat) and analyses were repeated in the per-protocol population, which included only *Shigella spp*. stool culture positive patient. In addition, the primary endpoint was analyzed in the subgroups defined by the following grouping criteria: culture positive vs. negative, *Shigella* pathogen vs. *Salmonella* pathogen and Nalidixic acid sensitive *Shigella* vs. Nalidixic acid resistant *Shigella*. Heterogeneity of the treatment effect was tested with a likelihood ratio test based on a logistic regression model that included an interaction between treatment effect and the sub-grouping criteria. P-values for two-sided tests and 95% confidence intervals are reported throughout. All analyses were performed with the statistical software R version 2.9.1 (R Foundation for Statistical Computing, Vienna, Austria, www.r-project.org.)

## Results

### Study participant flow

**Figure 1:**
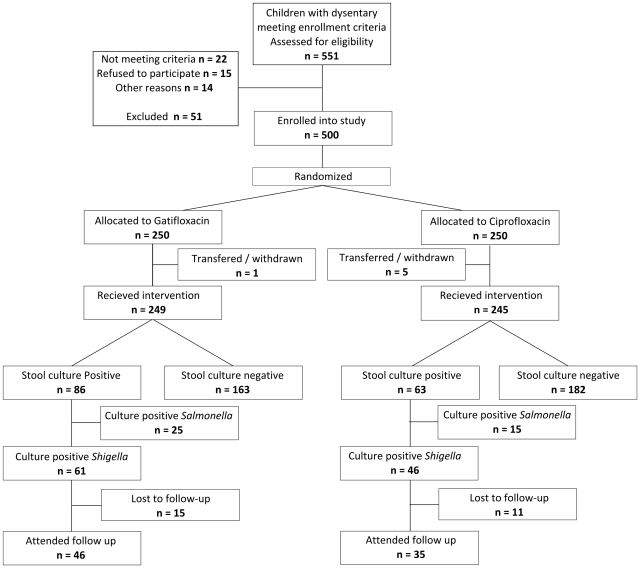
Profile for the gatifloxacin versus ciprofloxacin shigellosis treatment trial. The CONSORT flow diagram showing the flow of participants throughout the trial.

Between June 22^nd^, 2006 and March 13^th^, 2009 551 pediatric patients hospitalized with acute dysentery were assessed for enrolment in this RCT. The flow chart of the trial is presented in Figure 1, and shows that out of the eventual 500 enrolled patients randomized (300 at Huu Nghi Hospital, Dong Thap and 200 at the Hospital for Tropical Diseases), 494 received at least one dose of either treatment, corresponding with 245 patients in the ciprofloxacin group and 249 patients in the gatifloxacin group.

### Baseline information

The baseline characteristics of the intention to treat patients (ITT) are shown in Table 1. The median age of the 494 patients in the intention-to-treat population (ITT) was 19 months, with a range of 2 to 144 months, 58.9% were Male. Patients had a median duration of dysentery prior to admission of 24 hours, with a range of 1 to 72 hours. The most common clinical observation was fever, as 87.4% of patients had a temperature > 37.8°C at randomization. Forty two point five percent (n  =  210) of patients were enrolled with a history of blood in their stools and 57.5% of enrollees (n  =  284) had a history of mucus without blood in their stools. Baseline characteristics, in particular disease severity (signified by blood in the stool, number of diarrheal episodes, fever, vomiting and abdominal pain), were comparable in both groups (Table 1).

**Table 1:** The baseline characteristics and clinical symptoms of 494 intention-to-treat patients whom received an intervention.

Patient Characteristic	All patients (n = 494)	Gatifloxacin (n = 249)	Ciprofloxacin (n = 245)
Details			
Age (months)	19 (10.1–32)	19 (10–31)	19 (11–33)
Male sex	291 (58.9%)	143 (57.4%)	148 (60.4%)
Weight (Kg)	10 (8.2–12)	10 (8.2–12)	10 (8.4–12)
Height (cm)	78.5 (70–88)	78 (70–87)	79 (70–89)
Nutritional status			
Overweight	4 (0.8%)	1 (0.4%)	3 (1.2%)
Normal	363 (73.5%)	187 (75.1%)	176 (71.8%)
Malnutrition I	93 (18.9%)	47 (18.9%)	46 (18.8%)
Malnutrition II	29 (5.9%)	13 (5.2%)	16 (6.5%)
Malnutrition III	5 (1%)	1 (0.4%)	4 (1.6%)
Clinical observations prior on admission			
Illness duration prior to admission (hrs)	24 (16.3–48)	24 (16–48)	24 (18–48)
Fever (≥37.8°C)	429 (87.4%)	215 (87.4%)	214 (87.4%)
History of febrile convulsions	40 (8.1%)	19 (7.6%)	21 (8.6%)
History of diarrhea with blood	210 (42.5%)	107 (43%)	103 (42%)
History of mucoid diarrhea without blood	284 (57.5%)	142 (57%)	142 (58%)
Vomiting	204 (41.3%)	91 (36.6%)	113 (46.1%)
Abdominal pain	365 (74.2%)	188 (75.8%)	177 (72.5%)
Tenesmus	339 (69.2%)	173 (70%)	166 (68.3%)
Clinical observations within 24 hours of admission			
Mucoid diarrhea without blood	370 (74.9%)	193 (77.5%)	177 (72.2%)
Mucoid Diarrhea (number in 24 hr period)	3 (0–6)	3 (1–6)	3 (0–6)
Diarrhea with blood	445 (90.1%)	224 (90%)	221 (90.2%)
Bloody Diarrhea (number in 24 hr period)	1 (1–5)	1 (1–5)	2 (1–5)
Maximum number of episodes in 24 hr period	6 (3–10)	6 (3–10)	6 (3–9)
White blood cells in stool 1			
0	214 (44.7%)	111 (45.1%)	103 (44.2%)
+1	79 (16.5%)	42 (17.1%)	37 (15.9%)
+2	42 (8.8%)	17 (6.9%)	25 (10.7%)
+3	104 (21.7%)	56 (22.8%)	48 (20.6%)
+4	40 (8.4%)	20 (8.1%)	20 (8.6%)
White blood cell count	11,290 (8,530–14,900)	11,100 (8,530–14,200)	11,450 (8,572–15,600)
Pathogen isolated			
*Shigella*	107 (21.7%)	61 (24.5%)	46 (18.8%)
Nalidixic acid resistant *Shigella*	72 (14.6%)	40 (16.1%)	32 (13.1%)
*Salmonella*	40 (8.1%)	25 (10%)	15 (6.1%)
Other	2 (0.4%)	0 (0%)	2 (0.8%)

Summary statistics are absolute counts (%) for categorical variables and medians (IQR) for continuous data.

1 White blood cells in stool 0; 0 cells/HPF, 1; 1 to 10 cells/HPF, 2; 11 to 20 cells/HPF, 3: 21 to 30 cells/HPF and 4; >30 cells/HPF.

There were more patients with a bacterial pathogen positive stool culture in the gatifloxacin group (n  =  86, 34.5%) than in the ciprofloxacin group (n  =  63, 25.7%), of which 61 and 46 were *Shigella spp.* respectively. Sixty seven point three percent (n  =  72) of the *Shigella* isolated were resistant to nalidixic acid (MIC ≥16 µg/ml), additionally, the same proportion (n  =  72) of *Shigella* isolates were *S. sonnei*, 33 were *S. flexneri* and 2 were *S. boydii*.

### Study endpoints and outcomes

Results for the primary and secondary endpoints in the ITT population are shown in Table 2. The primary endpoint (composite overall treatment failure), demonstrated that fifty-seven (11.5%) of all ITT patients failed treatment, of which, 30 were in gatifloxacin group and 27 were in ciprofloxacin group (*p*  =  0.72). The majority of patients that failed therapy had clinical failure, which was principally associated with persistent diarrhea for longer than 120 hours after initiating treatment. There were four patients with prolonged fever and nine with microbiological failures in the ciprofloxacin group and six microbiological failures in the gatifloxacin group. There were no significant difference in secondary endpoints related to time to resolution of symptoms (Table 2).

**Table 2:** Primary and secondary trial endpoints.

Parameter	All patients (n = 494) 1	Gatifloxacin (n = 249) 1	Ciprofloxacin (n = 245) 1	Comparison:Estimate (95% CI) 2	*p*-value 3
Primary endpoint					
Overall Treatment Failure	57 (11.5%)	30 (12.0%)	27 (11.0%)	1.0 (−4.7 to 6.7)	0.72
b) Clinical Failure	45 (9.1%)	26 (10.4%)	19 (7.8%)	2.7 (−2.5 to 7.8)	0.30
Symptom > 5 days failure	13 (2.6%)	8 (3.2%)	5 (2.0%)	1.2 (−1.8 to 4.2)	0.42
Fever failure	4 (0.8%)	0 (0%)	4 (1.6%)	−1.6 (3.5 to 0.3)	0.06
b) Microbiological Failure	15 (3.0%)	6 (2.4%)	9 (3.7%)	−1.3 (−4.5 to 1.9)	0.41
Secondary endpoints 4					
Fever clearance time (hrs)	12 (0–30)	12 (0–30)	12 (0–28)	1.00 (0.84 to 1.20)	0.98
Bloody diarrhea clearance time (hrs)	24 (17–48)	24 (17–47)	25 (17–48)	1.11 (0.93 to 1.32)	0.26
Diarrhea clearance time (hrs)	61 (40–90)	61 (41–92)	61 (38–90)	0.98 (0.82 to 1.17)	0.84
Total time of illness from study enrolment (hrs)	64 (42–92)	64 (42–93)	64 (41–90)	0.99 (0.83 to 1.18)	0.90
Total time of illness from illness onset (hrs)	95 (66–123)	95 (66–126)	93 (68–120)	0.98 (0.82 to 1.17)	0.83
Follow up					
Patients attending follow up (n)	432 (87.4%)	217 (87.1%)	215 (88.8%)	-	-
Diarrhea on follow up (n)	15 (3.5%)	8 (3.7%)	7 (3.3%)	0.4 (−3.2 to 4.1)	0.81
Other symptom on follow up (n)	2 (0.5%)	2 (0.9%)	0 (0%)	0.9 (−0.9 to 2.7)	0.51
Failure on follow up (n)	17 (3.9%)	10 (4. 6%)	7 (3.3%)	1.4 (−2.5 to 5.2)	0.47

1 Summarized as n (%) for proportion data and median (IQR) for time-to event data.

2 Estimate corresponds to the absolute risk difference (in%) for proportion data and a hazard ratio for time-to-event data.

3 *p-*values calculated by chi-squared test for proportional data and by Cox Regression models for time-to-event data.

4 Time-to-event endpoints calculated from study enrolment (i.e. the first dose of study treatment) unless mentioned otherwise.

In the per-protocol analysis (*Shigella spp*. stool culture positive patients), the risk of treatment failure was as 4/61 (6.6%) for gatifloxacin and 5/46 (10.9%) for ciprofloxacin (*p*  =  0.49). Three out of sixty one (4.9%) and 2/46 (4.3%) in the gatifloxacin group and the ciprofloxacin group, respectively, had persistent diarrhea for longer than 120 hours post treatment. In the per-protocol analysis, the time to resolution of symptoms was also similar in the gatifloxacin and ciprofloxacin groups, respectively: time from study enrolment to fever clearance (median 18 vs. 18 hours, *p*  =  0.45) or bloody diarrhea clearance (median 24 vs. 24 hours, *p*  =  0.74) and total illness duration (median duration from illness onset 77 vs. 71 hours, *p*  =  0.48). The risk of overall treatment failure in selected subgroups is displayed in Table 3, which shows no evidence of treatment effect heterogeneity.

**Table 3:** Overall treatment failure in subgroups.

Subgroup	Gatifloxacinfailures (n)	Ciprofloxacinfailures (n)	RD (95% CI), p-value 1	*p*-value forheterogeneity 2
Population				
Culture positiveCulture negative	17/86 (19.8%)13/163 (8.0%)	16/63 (25.4%)11/182 (6.0%)	−5.6 (−19.3 to 7.9); *p* = 0.411.9 (−3.6 to 7.5); *p* = 0.48	0.28
Pathogen 3				
*Shigella* *Salmonella*	4/61 (6.6%)13/25 (52.0%)	5/46 (10.9%)9/15 (60.0%)	−4.3 (−16.1 to 6.9); *p* = 0.50−8.0 (−37.0 to 23.1); *p* = 0.62	0.81
Nalidixic acid resistance 4				
Resistant *Shigella*Sensitive *Shigella*	2/40 (5.0%)2/20 (10.0%)	4/32 (12.5%)1/13 (7.7%)	−7.5 (−21.8 to 6.7); *p* = 0.392.3 (−22.1 to 22.7); *p* = 1.0	0.70

1 RD  =  absolute risk difference (%).

2 Heterogeneity was tested with a likelihood ratio test based on a logistic regression model that included an interaction between treatment effect and subgroup.

3 Two patients with *Pleisiomonas* and *Morganella* infections (both in the ciprofloxacin group) are not included.

4 Nalidixic acid resistance status was not available for two patients with *Shigella* infections.

The proportion of patients that attended the follow up health visit was 87.4%. None of the 17 (3.9%) patients attending follow up which still demonstrated evidence of disease had a *Shigella spp.* positive stool culture, yet 15 (3.5%) still had persistent diarrhea (Table 2). There was no significant difference in prolonged symptoms synonymous with dysentery between the two study groups in the ITT analysis or the PP analysis.

### Adverse events

There is a concern about the use of gatifloxacin, as it has been associated with dysglycemia (hypoglycemia and hyperglycemia) in elderly adults (both diabetic and non-diabetic) [27]. We compared the blood glucose levels to detect dysglycemia on day zero (prior to the first dose) (n  =  244) and on day three (blood sample taken on same time of day as the primary sample) (n  =  234) in patients in gatifloxacin group. On day zero, 180/244 (73.8%) had normal blood glucose (65–115 mg/dl), 49/244 (20%) had mild hyperglycemia (116–160 mg/dl), 6/244 (2.5%) had moderate hyperglycemia (161–250 mg/dl), 5/244 (2%) had mild hypoglycemia (55–64 mg/dl) and 4/244 (1.6%) had moderate hypoglycemia (40–54 mg/dl). On Day three, 193/234 (82.5%) had glucose within the normal range, 33/234 (14.1%) had mild hyperglycemia, 5/234 (2.1%) had moderate hyperglycemia and 3/234 (1.3%) had mild hypoglycemia. Paired glucose data were for 234 patients treated with gatifloxacin, the means of blood glucose levels on day zero and day three were 100.7 mg/dl and 98.4 mg/dl, respectively (*p*  =  0.18; 2 tailed t-test). There were limited number of non-severe events, these included, one patient in the gatifloxacin arm with night sweats and 18 children vomiting after taking the medication (12 in ciprofloxacin group and 6 in the gatifloxacin group). In the case of vomiting the treatment drug were re-administered one hour after the vomiting episode. There was no death or severe clinical complications during the course of the trial. The clinical status of two children (one in each group) after three days of the study treatments warranted the use of intravenous ceftriaxone.

## Discussion

Shigellosis remains a common infection in low and medium human index countries and in countries undergoing industrialization with areas of inadequate sanitation [28]. In the pediatric proportion of the population that suffers shigellosis an effective antimicrobial remains important for the management of infection. Despite shigellosis being self-limiting, antimicrobials are generally considered to be associated with clinical improvement and a shortened duration of disease. The findings of a recent Cochrane review support antimicrobial use in *Shigella* infections, although the overall strength of data on antimicrobial therapy for shigellosis is weak and requires further consideration [29]. One potential effect of a suitable antimicrobial therapy for all *Shigella* patients is reducing fecal carriage of the organisms after infection, thus reducing transmission to close contacts, such as household members or children attending the same day-care setting [7], [23], [24].

A three day course of ciprofloxacin is the treatment currently recommended by the WHO for shigellosis, including *S. dysenteriae* type 1 infections [13]. In the study presented here, 494 children hospitalized for acute dysentery (in which 107 cases had a *Shigella* positive stool culture) were treated by either current WHO recommendations, or three days of one daily dose of oral gatifloxacin. Our study could not demonstrate the superiority of gatifloxacin. Rather, it showed a similar efficacy of both drugs in the treatment of childhood dysentery, including those with a stool culture confirmed *Shigella* infection. However, gatifloxacin has a longer half-life than ciprofloxacin and the once-a-day administration may be considered a little more convenient than twice-a-day regime of ciprofloxacin. In total, 67.3% of *Shigella* strains isolated were nalidixic acid resistant, but both antimicrobials appeared to clear *Shigella* from stools equally effectively (as tested by microbiological culture).

Our work has some caveats, firstly, blinding was not performed due to financial limitations and differing regimens, thus there was the potential of introducing investigator bias, yet, this was minimal due as result of the randomization process. Secondly, the primary endpoint was a composite endpoint of both clinical symptoms and microbiological failure, which was deemed more appropriate due to the nature of the infection. Furthermore, it was not possible to identify an infecting agent in the majority of cases, and we know from our currently unpublished work that more than one etiological agent may cause the infection. Therefore, whilst cessation of symptoms is the most relevant clinical end point (for the treating clinician and the patient) for shigellosis, it may be difficult to assess accurately and consistently. We cannot rule out that the persistence of symptoms may be caused by an additional infecting pathogen. Finally, it would be difficult to conduct such a trial in the community in this setting, etiological diagnostics for diarrheal disease are not routinely performed in this location and we are uncertain of the burden of shigellosis in the community. Our study may represent the most severe end of the disease spectrum for shigellosis, yet it is these individuals that may require the most apposite antimicrobial therapy.

Our data show similar overall risks of treatment failure in the two treatment groups (11% in the ciprofloxacin group versus 12% in gatifloxacin group). The overall risk of treatment failure rate of 8.4% in culture confirmed shigellosis cases is considerably lower than the 31% to 35% reported in the treatment of dysentery in a 2002 study [30]. This disparity in failure rate may be reflected by a difference in the principal *Shigella* serotype isolated. The most commonly isolated *Shigella* species here was *S. sonnei,* whilst *S. dysenteriae* was the most common isolated in the aforementioned 2002 study. *S. dysenteriae* causes a considerably more severe syndrome than *S. sonnei*, which is largely associated with the secretion of shiga toxin [31]. In an additional shigellosis treatment trial, the failure rate in adults treated with ciprofloxacin was 18% [32]. In dysentery treatment trial conducted in Ho Chi Minh City, *S. flexneri* was the dominant serogroup and the failure rate with the fluoroquinolone, ofloxacin, was comparable to our current data, at 10% [24]. A possible explanation for a reduced treatment failure rate, despite a pattern of reduced susceptibility to fluoroquinolones, is that shigellosis caused by *S. sonnei* is gradually becoming a more benign infection, an opinion that is shared by others [33]. Alternatively, due to improvements in education and healthcare, patients are now admitted to hospital earlier. Therefore, case management has improved and children receive therapy more rapidly after the onset of symptoms. Here, we observed similar failure rates in both treatment groups, despite a relationship between nalidixic acid resistance and reduced susceptibility to ciprofloxacin. These data suggest that whilst there has been a notable increase in MIC to nalidixic acid in *Shigella* in Vietnam over the last ten years, it may not yet be substantial enough to hinder the bactericidal effect of ciprofloxacin *in vivo*. A similar effect of both antimicrobial agents, despite gatifloxacin having greater *in vivo* activity supports the theory of a less severe infection, which may not, in all cases, require an antimicrobial for the cessation of symptoms. Alternatively, *Shigella* may respond in atypical manner to gatifloxacin, which respect to other Gram-negative organisms, and mutations in the *gyrA* and *parC* gene may have a greater effect on reducing the potency of the antimicrobial agent.

The use of fluoroquinolones in children has been contraindicated for many years because of the fear of arthropathy, however there is limited evidence to support this theory. A two year of follow-up of children treated with a short course ciprofloxacin or ofloxacin for typhoid fever in Vietnam did not find any adverse effects on growth and there was no evidence or arthropathy [34]. A more recent study conducted in a sheep model showed that neither ciprofloxacin nor gatifloxacin affected growth velocity when administered with using dosing regimen suitable for children [35]. The WHO, after considering the risks and the benefits, has recommended ciprofloxacin as the primary treatment of shigellosis in adults and children [13], our data suggests that this choice remains effective and we consider fluoroquinolones safe for use in the pediatric population. Gatifloxacin is an effective treatment against many Gram-negative infections, yet was voluntarily withdrawn from the US and Canadian drug market in May 2006 because of concerns about severe glucose disturbances. Reports of gatifloxacin related hypoglycemia and hyperglycemia in clinical trials are rare, yet severe dysglycemic events (mainly involved elderly patients with diabetes) were reported and discussed in the medical literature during the post-marketing period [27], [36], [37]. To date, clinically relevant dysglycemia has not been reported in young adults or in children treated with gatifloxacin [38], [39], we found no evidence of dysglycemia in the gatifloxacin treatment group. Treatment options for many Gram-negative infections in developing countries, including *Shigella* are clearly becoming increasingly limited. Gatifloxacin is a highly efficacious antimicrobial for the treatment of many gastrointestinal infections in the young and otherwise healthy patients and should be available for such indications in locations (i.e. in industrializing countries) where these diseases are endemic. However, in view of the potential risk of dysglycemia, it may be prudent to not treat patients over 50 years of age or with additional co-morbidities with such infections with gatifloxacin.

Our data demonstrate that despite a substantial increase in the number of organisms demonstrating resistance to nalidixic acid in the preceding years, ciprofloxacin remains an effective therapy for acute bacterial dysentery. Furthermore, we demonstrate that gatifloxacin is similarly effective as ciprofloxacin in the cessation of symptoms of shigellosis. Our current knowledge of the mechanisms and the global distribution of *Shigella* with reduced susceptibility to older fluoroquinolones (ciprofloxacin and ofloxacin) remain limited and require further investigation. We conclude that in locations where nalidixic acid resistant *Shigellae* are highly prevalent, ciprofloxacin and gatifloxacin are similarly effective for the treatment of acute shigellosis.

## Supporting Information

Checklist S1 CONSORT Checklist
(DOC)

Protocol S1 Trial Protocol
(PDF)
