# Supplementary material for: A Multi-Center Randomized Trial to Assess the Efficacy of Gatifloxacin versus Ciprofloxacin for the Treatment of Shigellosis in Vietnamese Children
Source: PLoS Negl Trop Dis. 2011 Aug 2;5(8):e1264. doi: 10.1371/journal.pntd.0001264 (PMC3149021; doi:10.1371/journal.pntd.0001264)
Supplement: Protocol S1 — Trial Protocol (PDF) [file pntd.0001264.s002.pdf]

The treatment of shigellosis in children: Comparison of ciprofloxacin and gatifloxacin

EG Study. Final Protocol. Version 3.

Authors. Dr Ha Vinh and Prof. Jeremy Farrar

May 2005

Controlled trials number ISRCTN55945881.

Investigators

Paediatric Ward B, Hospital for Tropical Diseases, Ho Chi Minh City, Vietnam

Department of Infectious Diseases, Huu Nghi Hospital, Cao Lanh, Dong Thap, Vietnam

Microbiology Laboratory, Hospital for Tropical Diseases, Ho Chi Minh City, Vietnam

Microbiology Laboratory, Huu Nghi Hospital, Cao Lanh, Dong Thap Vietnam

Oxford University Clinical Research Unit, Hospital for Tropical Diseases, Ho Chi Minh City, Vietnam

Background

Shigella cause 10-15% of acute diarrheal infections in children under 5 years and are the most common cause of bloody diarrhoea in children. Antimicrobial resistance of *Shigella spp*. to commonly used antibiotics, such as ampicillin and trimethoprim-sulfamethoxazole (TMP-SMX), has been reported from many countries. Nalidixic acid had been used successfully in these cases, but became ineffective because of the emergence of nalidixic acid resistance. Many fluoroquinolones antimicrobials (namely ciprofloxacin, norfloxacin and ofloxacin) have been proved effective in the treatment of shigellosis caused by nalidixic acid resistant strains. In Vietnam 62% of Shigella strains were resistant to multiple antibiotics , and ofloxacin is preferable to nalidixic acid in the treatment of bacillary dysentery [1]. In 2004, The World Health Organism organised a meeting of experts around the world and recommended ciprofloxacin or other fluoroquinolones should be used to treat shigellosis in adults and children. However, after being used for some years to treat shigellosis, the clinical response to ciprofloxacin treatment have decreased, strains of *Shigella dysenteriae* type 1 resist to ciprofloxacin were also detected. Searching for alternative regimens is an obviously necessity.

Gatifloxacin is a new 8-methoxy-fluoroquinolone antimicrobial. It has a broad spectrum of activity, with potent activity against gram-positive bacteria as well as significant activity against gram-negative and atypical organisms. The drug is distributed extensively into tissues. The MIC_{90} for *Shigella spp*. is 0.01-0.03mg/L . Gatifloxacin actively penetrates into phagocytic cells *in vitro*. Its significant accumulation in these cells (intracellular:extracellular concentration ratios 5-7 in macrophages and neutrophils) may result in enhanced activity against susceptible intracellular pathogens [2]. Gatifloxacin has been used to treat typhoid fever in adult and children with favourable results in Vietnam (Jeremy, F. unpublished data).

This study will compare the effectiveness of 3 days of ciprofloxacin with 3 days of gatifloxacin in the treatment of shigellosis in children.

Study Design

An open label randomized comparison of gatifloxacin for 3 days versus ciprofloxacin for 3 days for the treatment of uncomplicated bacillary dysentery in children. This trial is designed to demonstrate the superiority of gatifloxacin over ciprofloxacin.

Patient Selection

Entry criteria:

Patients admitted to Paediatric Ward B at Hospital for Tropical Diseases or Department of Infectious Diseases at Huu Nghi Hospital, Dong Thap in Vietnam. Patients considered for enrolment must be, < 15 years old, have a history of passing bloody stools or mucoid stools with/without abdominal pain or tenesmus with fever (>37.8^{o}C) an illness lasting less than 72 hours and the child’s parents must provide informed consent.

Exclusion criteria:

Any signs of a severe infection (shock, jaundice, extensive gastrointestinal bleeding), a history of hypersensitivity to either of the trial drugs, known previous treatment with any (fluoro)quinolone during the current disease prior to hospital admission or a coexisting infection requiring antimicrobial therapy and infection with a trophozoite or *Entamoeba histolytica* in their stool on microscopic examination.

Treatment

All the patients entered in the study will randomly receive one of two regimens. Randomization will be in blocks of 10 by and performed by an individual not involved in the trial. Allocations will be in sealed envelopes, opened after the patient will be enrolled into the trial. The two trial arms will be.

Group A: Ciprofloxacin (Bayer) 30 mg/kg/d in two divided doses for 3 days.

Group B: Gatifloxacin (Bristol-Myers Sqibb) 10 mg/kg/day in a single daily dose for 3 days.

Additional treatment in both groups (when required) will include, fluid (ORS, parenteral), antipyretics (paracetamol) and other treatments as indicated by the clinical situation. Seizures will be treated with diazepam 0.25 mg/kg intravenously. No additional anti-diarrhoeal drugs will be permitted.

Clinical Methods

1. Study day 0 will begin with the first administration of study drugs and continue for the next 24 hours.
2. A history of the present illness will be documented on a report form.
3. The clinical history and examination will be documented and will include; axillary temperature, pulse rate, respiratory rate, blood pressure, and frequency and stools characteristics (bloody, mucoid or watery) (these reading will be recorded ever six hours).
4. A full blood count will be performed on day 0 and each day of the study.
5. CRP will performed on Day 0.
6. A glucose test will be performed on day 0 and on day 3 on those in the gatifloxacin group

7. Patents will be required to attend a seven day follow up, for stool culture, temperature reading and a clinical interview about post-treatment illness.

Microbiology methods

1. Stool will be examined by microscopy for white cells, red cells, ova, cysts and parasites
2. A stool sample will be cultured daily for *Shigella* and *Salmonella* on entry to the study [D0] before antimicrobial treatment and for the next 3 consecutive days [D1,2,3] or until diarrhoea ceases.

Treatment response

1. Clinical failure rate
2. Fever clearance time: time for the axillary body temperature to fall below 37.8°C for 48 hours.
3. Diarrhoea cessation time: time to return to formed stools
4. Bacteriological clearance time: time to eradicate the causative bacteria from the stools.

Treatment outcomes

Treatment failure is defined as clinical or microbiological treatment failure (or both). A clinical treatment failure will be fever ($\geq$ 37.8°C), or the persistence of the disease (bloody diarrhoea, watery diarrhoea, vomiting, abdominal pain or tenesmus) after day 5 of treatment. Treatment failures will be treated with ceftriaxone at the discretion of the treating physician. A microbiological treatment failure will be a positive stool culture after the completion of the treatment. A cure will be cessation of all symptoms and signs by the fifth day of treatment. Time to cessation of individual symptoms will be recorded and will include, fever clearance time (FCT) (time until the temperature $\leq$ 37.8°C and remains at this level for 48 hours; bloody diarrhea clearance time (BDCT), (time until the first non-bloody stool recorded) and diarrhoea clearance time (DCT).

Sample size

For 80 % power and 5 % significance, each group will need 58 culture confirmed children in order to detect a difference of 20 % in the clinical cure rate between ciprofloxacin (est .75 %) and gatifloxacin (est 95 %). Because the rate of positive stools culture for *Shigella* is approximately 33 % of bloody diarrhoea, and the drop-out rate will be about 5 %, We need to enrol 366 cases. An interim analysis will be performed after every 60 completed cases.

Time to begin and duration of study

The study will begin after the approval of the Scientific and Ethical Committee [Jan 2006], and will last for approximately 2 years.

Supply

Antimicrobial and laboratory support will be provided by The Oxford Univesrity Clinical Research Unit.

Ethical Approval

This study will require the approval of The Scientific and Ethical Committee of The Hospital for Tropical Diseases, Ho Chi Minh City, Vietnam and The Oxford Tropical Research Ethics Committee United Kingdom.

References

1. Vinh H, Wain J, Chinh MT, et al. Treatment of bacillary dysentery in Vietnamese children: two doses of ofloxacin versus 5-days nalidixic acid. Trans R Soc Trop Med Hyg 2000;94(3):323-6.

2.Douglas N. Fish. Gatifloxacin, an Advanced 8-Methoxy Fluoroquinolone. Pharmacotherapy 2001;21(1):35-59
